# Supplementary material for: Identification of miRNAs in Response to Sweet Potato Weevil (Cylas formicarius) Infection by sRNA Sequencing
Source: Genes (Basel). 2022 May 30;13(6):981. doi: 10.3390/genes13060981 (PMC9222952; doi:10.3390/genes13060981)
Supplement: Supplementary file 1 [file genes-13-00981-s001.zip › Figure S1. GO and KEGG analysis of target genes for differentially expressed miRNAs..pdf]

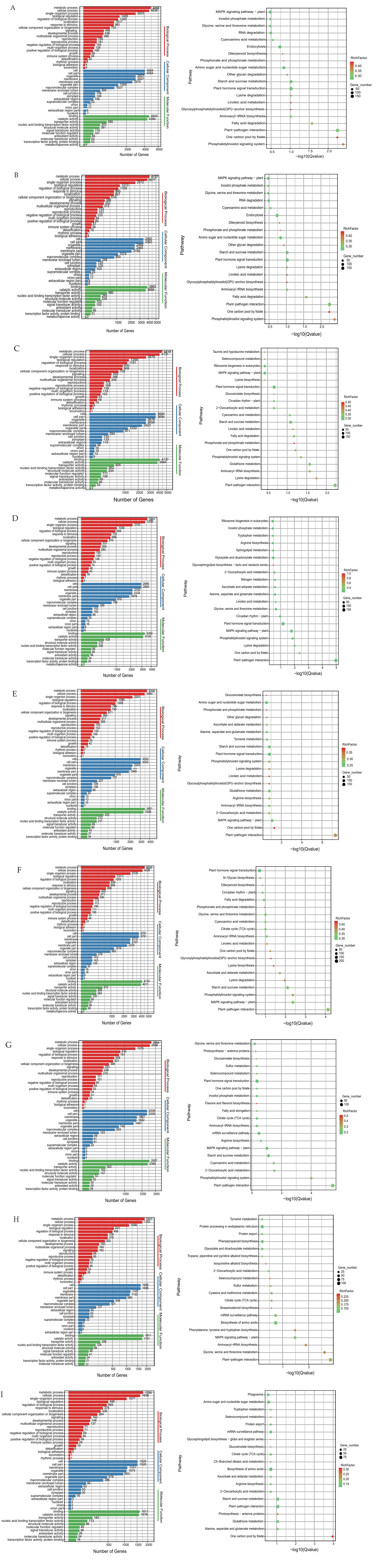

Supplementary Figure S1 GO and KEGG analysis of target genes for differentially expressed miRNAs.
(A) E0 vs E24 , Left: GO analysis; Right: KEGG analysis; (B) E0 vs E48 , Left: GO analysis; Right: KEGG analysis; (C) E0 vs E72 , Left: GO analysis; Right: KEGG analysis; (D) G0 vs G24 , Left: GO analysis; Right: KEGG analysis; (E) G0 vs G48 , Left: GO analysis; Right: KEGG analysis; (F) G0 vs G72 , Left: GO analysis; Right: KEGG analysis; (G) E24 vs G24 , Left: GO analysis; Right: KEGG analysis; (H) E48 vs G48 , Left: GO analysis; Right: KEGG analysis; (I) E72 vs G72 , Left: GO analysis; Right: KEGG analysis.
